# Multiple sulfatase deficiency with neonatal manifestation

**DOI:** 10.1186/s13052-014-0086-2

**Published:** 2014-12-17

**Authors:** Livia Garavelli, Lucia Santoro, Alexandra Iori, Giancarlo Gargano, Silvia Braibanti, Simona Pedori, Nives Melli, Daniele Frattini, Lucia Zampini, Tiziana Galeazzi, Lucia Padella, Stefano Pepe, Anita Wischmeijer, Simonetta Rosato, Ivan Ivanovski, Lorenzo Iughetti, Chiara Gelmini, Sergio Bernasconi, Andrea Superti-Furga, Andrea Ballabio, Orazio Gabrielli

**Affiliations:** Clinical Genetics Unit, Obstetric and Pediatric Department, Istituto di Ricovero e Cura a Carattere Scientifico, Arcispedale Santa Maria Nuova, Reggio Emilia, Italy; Pediatrics Unit, UNIVPM, Ancona, Italy; Department of Medical and Surgical Sciences of Childhood and Adult, University of Modena and Reggio Emilia, Modena, Italy; Neonatal Intensive Care Unit, Obstetric and Pediatric Department, Istituto di Ricovero e Cura a Carattere Scientifico, Arcispedale Santa Maria Nuova, Reggio Emilia, Italy; Pediatric Neurology Unit, Obstetric and Pediatric Department, Istituto di Ricovero e Cura a Carattere Scientifico, Arcispedale Santa Maria Nuova, Reggio Emilia, Italy; Telethon Institute of Genetics and Medicine (TIGEM), Via Pietro Castellino 111, 80131 Naples, Italy; Department of Medical Genetics, Policlinico Sant’Orsola-Malpighi, University of Bologna, Bologna, Italy; Deparment of Pediatrics, University of Parma, Parma, Italy; Department of Pediatrics, Centre Hospitalier Universitaire Vaudois, University of Lausanne, Lausanne, Switzerland; Department of Molecular and Human Genetics, Baylor College of Medicine, Houston, TX 77030 USA; Jan and Dan Duncan Neurological Research Institute, Texas Children Hospital, Houston, TX 77030 USA; Medical Genetics, Department of Translational Medicine, Federico II University, Via Pansini 5, 80131 Naples, Italy

**Keywords:** Multiple sulfatase deficiency, MSD, *SUMF1* gene

## Abstract

Multiple Sulfatase Deficiency (MSD; OMIM 272200) is a rare autosomal recessive inborn error of metabolism caused by mutations in the sulfatase modifying factor 1 gene, encoding the formylglycine-generating enzyme (FGE), and resulting in tissue accumulation of sulfatides, sulphated glycosaminoglycans, sphingolipids and steroid sulfates. Less than 50 cases have been published so far. We report a new case of MSD presenting in the newborn period with hypotonia, apnoea, cyanosis and rolling eyes, hepato-splenomegaly and deafness. This patient was compound heterozygous for two so far undescribed SUMF1 mutations (c.191C > A; p.S64X and c.818A > G; p.D273G).

## Introduction

Multiple Sulfatase Deficiency is a rare autosomal recessive inborn error of metabolism characterized by the defective activity of all known sulfatases [1]. It is caused by mutations in the sulfatases- modifying factor 1 gene encoding the FGE. Sulfatases are a family of enzymes that catalyze the hydrolysis of ester sulfates, including glycosaminoglycans, sulfolipids and steroid sulfates. These proteins share high amino acid sequence homology. However they show different sub-cellular localization and substrate specificity [1,2]. The *SUMF1* gene encodes for a sulfatase-modifyng factor, which converts a highly conserved cysteine within the sulfatase catalytic domain into Cα- formylglycine [3]. It has been shown that this post-translational modification is both an essential and limiting factor for the enzymatic activity of sulfatases [4]. The *SUMF1* gene is located on chromosome 3p26. It spans 105 kb and the coding sequence is distributed over 9 exons. The cDNA for human FGE is predicted to encode a protein of 374 residues. The protein contains a cleavable signal sequence of 33 residues, which indicates translocation of FGE into the endoplasmatic reticulum, and contains a single N-glycosylation site at Asn-141 [5]. The middle part of FGE (residues 179–308 in human FGE) is represented by a tryptophan-rich subdomain. The C-terminal subdomain (residues 327–366 in human FGE) is the most highly conserved sequence within the FGE family.

FGE post translationally activates all newly synthesized sulfatase by generating the catalytic residue formylglycine. Impaired FGE function leads to a reduction in sulfatase activities. It has been proved that variability of the clinical phenotype depends on both residual FGE activity as well as protein stability [6]. MSD is characterized by features of mucoplysaccharidosis and metachromatic leukodystrophy including neurologic deterioration and development delay, gargoyle-like features, visceromegaly, heart involvement, ichthyosis, hydrocephaly, cloudy corneal and skeletal anomalies [7]. According to the age of onset, very neonatal, late infantile (severe or LIS and mild or LIM) and rare juvenile (mild) disease subtypes can be distinguished [8].

We described a clinical case of a girl with a neonatal form of MSD, presenting typical findings of the disease. She was compound heterozygous for two so far undescribed *SUMF1* mutations (c.191C > A; p.S64X and c.818A > G; p.D273G).

## Clinical report

**Figure 1:**
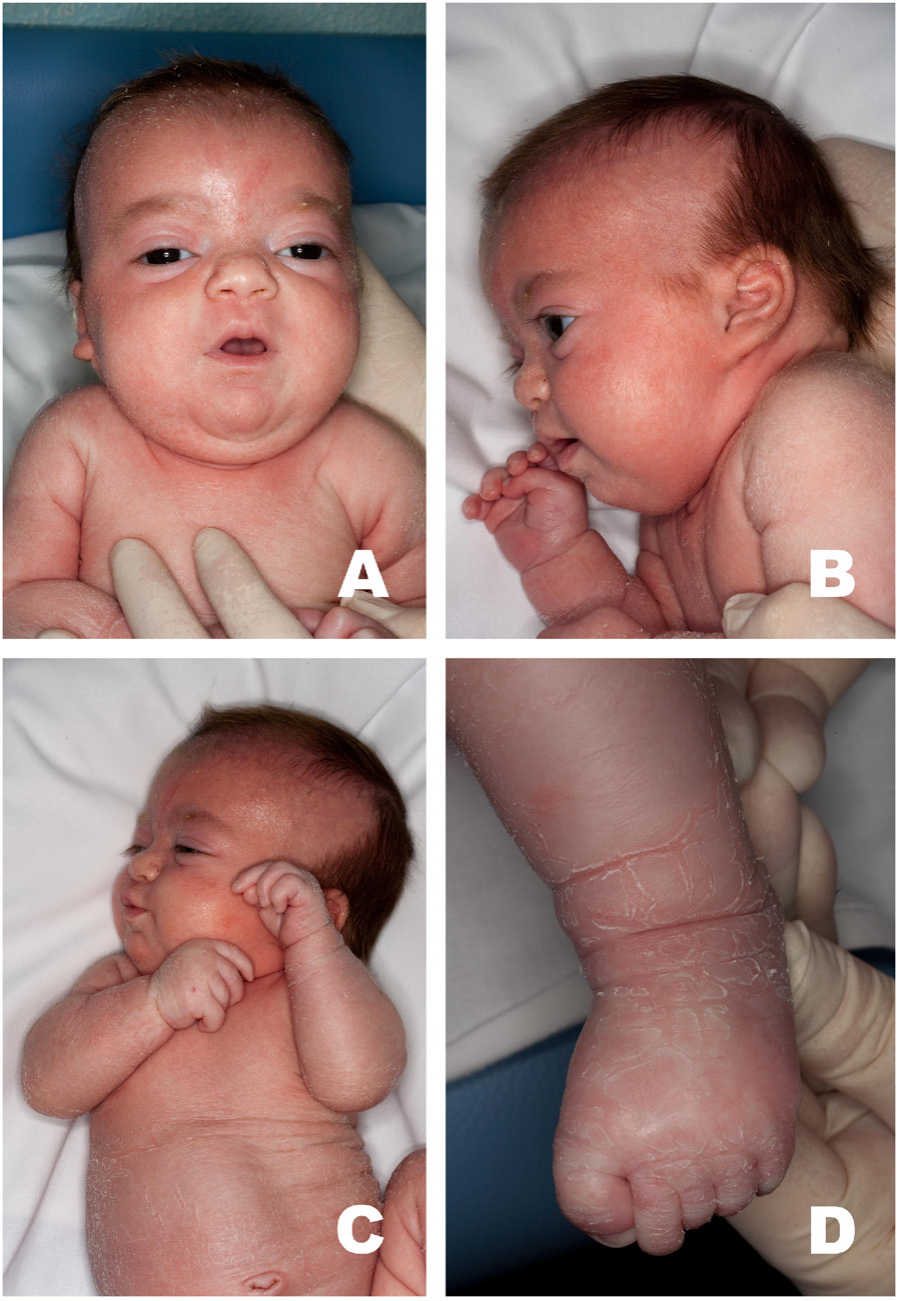
**Phenotype. A**: coarse face, high-arched, thick eyebrows, bulbous nasal tip and mild ichthyosis. **B**: micrognathia, posteriorly rotated ears with attached lobes. **C**: abdominal ichthyosis. **D**: ichthyosis of the lower limb and feet.

The girl is the first child of healthy, non-consanguineous parents. She was born by vaginal delivery at week 40 with birth weight of 2,815 g, length 46 cm, and head circumference 32 cm. APGAR scores were 9/10. At 20 hours of life she was transferred to our Neonatal Intensive Unit because of the appearance of rolling eyes followed by episodes of apnoea with cyanosis and hypotonia, for which a treatment with phenobarbital was installed. At that time, physical examination showed coarse facial features, high-arched, thick eyebrows, bulbous nasal tip, micrognathia, posteriorly rotated ears with attached lobes, flared thorax, inverted nipples, hepatosplenomegaly, broad thumbs, mild ichthyosis and muscular hypotonia. (Figure 1A, B, C, D). At 11 days of life she presented two episodes of obstructive respiratory arrest after stridor, treated with ventilation. The otolaryngologist’s evaluation revealed the presence of a cystic formation of the vallecula, which was removed surgically with resolution of the episodes of apnoea.

Because of the coarse facial features, hypertrichosis, and hepatosplenomegaly, a lysosomal storage disorder was considered. There was an increased urinary excretion of glycosaminoglycans (1101 μg/mg creatinine; normal range, 30–200), with chondroitin sulfate (+++), dermatan sulfate (+++) and heparan sulfate (++) on electrophoresis. Enzyme activitiy testing in leucocytes showed deficiency of arylsulfatase A (0.0 nM/mg/h; n.v. 40–270), arylsulfatase B (0.0 nM/mg/h; n.v. 70–126), iduronate sulfatase (0.9 nM/mg/4 h; n.v. 51–123) and galactose 6 sulfatase (0.6 nM/mg/17 h; n.v.20-27). This pattern was diagnostic of Multiple sulfatase deficiency (MSD). Molecular analysis of the gene *SUMF1* revealed two so far undescribed heterozygous mutations: c.191C > A s. S64X in exon 1 and c.818A > G p.D273G in exon 6. The first heterozygous mutation was also demonstrated in the girl’s mother, while the second one was identified in the girl’s father.

**Figure 2:**
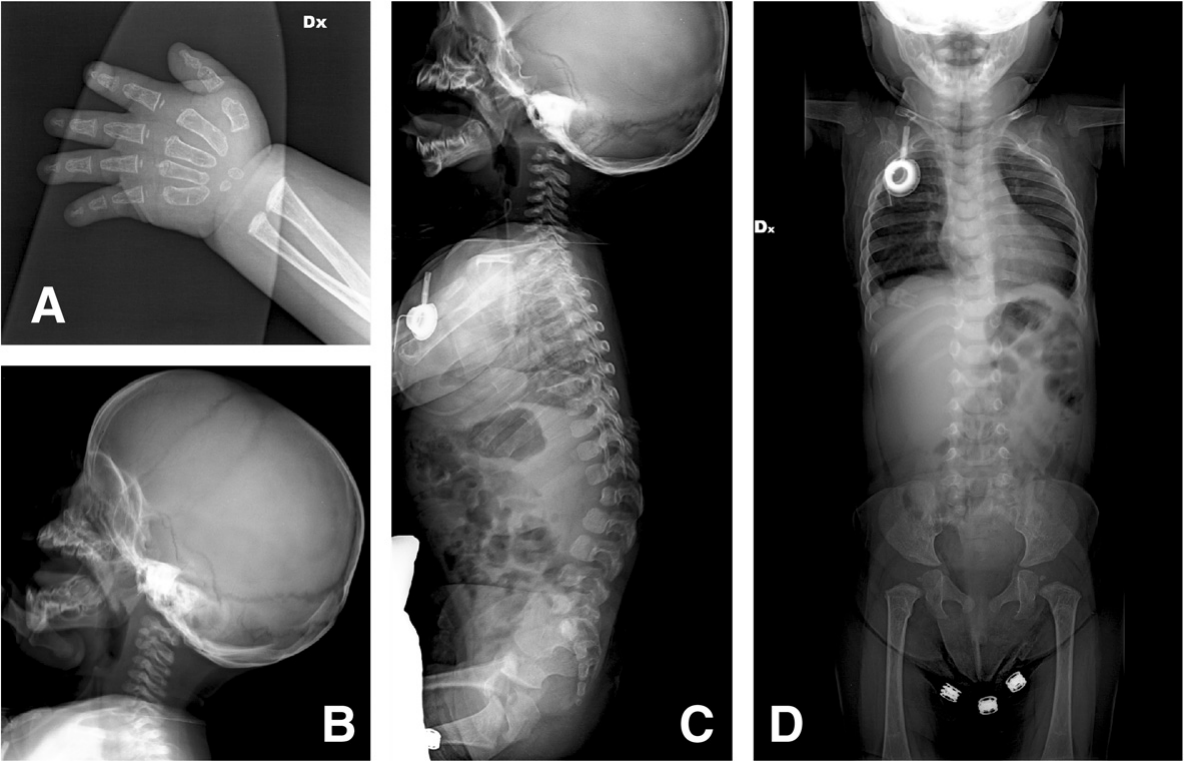
**X-Rays. A**: broad and hypoplastic first metacarpals, pointing of the second and third metacarpals, irregularity of the distal radial and ulnar metaphysis. **B**: platyspondily of the cervical vertebral bodies. **C**: hypoplastic vertebral body of L2, lumbar kyphosis. **D**: broad ribs.

The karyotype was normal, 46,XX. The echocardiography and EEG were normal; abdominal ultrasound showed hepatomegaly; brain magnetic resonance imaging (MRI) showed dilated lateral ventricles with an enlarged cisterna magna, demyelination of the white matter, partial corpus callosum hypoplasia with hypothrophy of the splenium and dismorphism of the hippocampum. Skeletal radiography revealed a pattern of dysostosis multiplex with hypoplasia of L2, lumbar kyphosis and platyspondyly of the cervical vertebral bodies; broad ribs; broad and hypoplastic first metacarpals, pointing of the second and third metacarpals, irregularity of the distal radial and ulnar metaphysis (Figure 2A, B, C, D). The ABR examination showed mild hypoacusia.

The child presented global developmental delay: could hold head up only at the age of 10 months, at the age of 12 months she was still hypotonic, with limited and jerky movements and it was impossible to meet her gaze. She developed dorso-lumbar kyphosis and owing to L2 hypoplasia she had to wear a orthopaedic corset.

She was seen again in our Clinical Genetics Unit at 24 months of age. Her head circumference was 45 cm (50th centile), length 66 cm (<3rd centile), and weight 6.406 Kg (<<3rd centile). Her psychomotor development was severely delayed and she uttered no words. At the age of 24 months she could not sit up unaided.

## Discussion

Multiple sulfatase deficiency is a rare autosomal recessive inborn error of metabolism affecting post-translational activation of sulfatases by the formylglycine generating enzyme (FGE). Due to mutations in the encoding *SUMF1* gene, FGE’s catalytic capacity is impaired, resulting in reduced cellular sulfatase activities [9]. More than 30 different *SUMF1* mutations are known, most of them missense mutations that affect stability and residual molecular activity of mutant FGE, which both determine MSD disease severity [10]. A variable residual activity of the different sulfatases has been described [1,11].

Few studies were able to show a correlation of both residual activity and stability of FGE variants with the clinical presentation of selected MSD patients. Patients with drastic impairments of both FGE stability and residual enzyme activity displayed the most severe clinical phenotype whereas the mildest phenotype was associated with the highest residual FGE activity among the studied variants.

It is now possible to establish a genotype/phenotype correlation for MSD and to roughly predict the clinical course for patients with the studied *SUMF1* mutations. Missense mutations affecting crucial functional or structural residues in FGE, as well as nonsense ones, will cause severe forms of the disease, whereas missense mutations not fully abrogating a functional conformation of the FGE protein will lead to attenuated forms [6].

Different types of MSD can be distinguished according to the age of onset: neonatal, late infantile (0 to 2 years, severe or LIS and mild or LIM), and rare juvenile (2 to 4 years, mild) [8].

The clinical picture of MSD combines symptoms of the different sulfatase deficiencies. Patients show neurological deterioration and a neurodegenerative course of disease similar to metachromatic leukodystrophy. Development delay, dysmorphism and organomegaly are present as found in various mucopolysaccharidoses [6]. Skeletal abnormalities remind one of Chondrodysplasia punctata type 1 and skin changes of X-linked ichthyosis [1].

The onset of symptoms in early infancy is associated with poor chances of survival; conversely, when features of the disease appear in late infancy, patients survive longer.

In general it is rare to carry out a diagnosis of storage disease in newborns apart from I-cell disease also known as mucolipidosis II, which is a condition with a severe clinical course, typical radiological aspects and fibroblast inclusions. Other symptoms include hyperplastic gums, thoracic deformities and congenital hip dislocation which are evident in newborn age. In the other storage diseases the symptoms are not so evident in the newborn age.

The diagnosis of MSD may not be simple due to the rarity of the condition: in our case the features which led us to suspect the diagnosis were the MPS-like phenotype, the radiological signs compatible with “dysostosis multiplex” and especially the cutaneous features. The child had no signs of chondrodysplasia punctata, in particular no epiphyseal stippling.

Indeed the presence of ichthyosis can be a very useful indicator in identifying the condition and can be the best marker of the disease in newborns.

Our patient had typical features of neonatal MSD including neonatal hypotonia, coarse face, mild deafness, visceromegaly, hypoplastic vertebral bodies and delayed psychomotor development.

It was demonstrated that the phenotypic outcome depends on both residual FGE activity as well as protein stability [6]. Another neonatal case was described which showed all the clinical symptoms of the condition and a quick worsening course of the disease: he presented two nonsense mutations leading to almost fully abrogated FGE activity, highly unstable FGE protein and nearly undetectable sulfatase activity [6]. Our case presents a missense mutation along with a nonsense mutation.

In fact we have identified two so far undescribed SUMF1 mutations (c.191C > A; p.S64X and c.818A > G; p.D273G), resulting in a severe clinical phenotype with early onset, but which allow survival beyond one year of life, in contrast with what has been reported in literature up to this point. She has a serious clinical aspect, but probably less severe than that described by Schlotawa and is still alive at the age of 2 years.

MSD is still an untreatable disease. Thus, potential treatment approaches, and also genetic counselling, directly depend on thorough analyses of the functional consequences of human *SUMF1* mutations on the clinical and biochemical phenotype.

Understanding better the molecular mechanism at the root of this condition could aid the identification of the most appropriate therapeutic approach of MSD in the near future.

## Conclusion

In conclusion, our patient illustrates well the main clinical features to suspect this condition in the newborn age, ichthyosis in particular, the wide spectrum of clinical manifestations and biochemical abnormalities of this rare disease. This case serves to highlight the importance of full and detailed evaluation of all patients with rare disorders and the need for continued updates of suggested surveillance for these diseases.

## Consent

Written informed consent was obtained from the patient’s guardian/parent/next of kin for the publication of this report and any accompanying images”.
